# Identification of SLC25A46 interaction interfaces with mitochondrial membrane fusogens Opa1 and Mfn2

**DOI:** 10.1016/j.jbc.2024.107740

**Published:** 2024-08-31

**Authors:** Sivakumar Boopathy, Bridget E. Luce, Camila Makhlouta Lugo, Pusparanee Hakim, Julie McDonald, Ha Lin Kim, Jackeline Ponce, Beatrix M. Ueberheide, Luke H. Chao

**Affiliations:** 1 Department of Molecular Biology, Massachusetts General Hospital, Boston Massachusetts, USA; 2 Department of Genetics, Harvard Medical School, Boston Massachusetts, USA; 3 Proteomics Resource Center, Division of Advanced Research Technologies, New York University Langone Health Center, New York New York, USA; 4 Department of Biochemistry and Molecular Pharmacology, New York University Langone Health Center, New York New York, USA

**Keywords:** mitochondria, membrane fusion, GTPase, structural model, protein cross-linking, mass spectrometry, protein-protein interaction, mitochondrial solute carrier

## Abstract

Mitochondrial fusion requires the sequential merger of four bilayers to two. The outer-membrane solute carrier family 25 member (SLC25A46) interacts with both the outer and inner membrane dynamin family GTPases mitofusin 1/2 and optic atrophy 1 (Opa1). While SLC25A46 levels are known to affect mitochondrial morphology, how SLC25A46 interacts with mitofusin 1/2 and Opa1 to regulate membrane fusion is not understood. In this study, we use crosslinking mass spectrometry and AlphaFold 2 modeling to identify interfaces mediating an SLC25A46 interaction with Opa1 and Mfn2. We reveal that the bundle signaling element of Opa1 interacts with SLC25A46, and present evidence of an Mfn2 interaction involving the SLC25A46 cytosolic face. We validate these newly identified interaction interfaces and show that they play a role in mitochondrial network maintenance.

Mitochondrial fission and fusion are membrane remodeling processes required for maintenance of a healthy mitochondrial reticulum (1, 2), which regulates the number and distribution of mitochondrial DNA and respiratory chain proteins (1, 2, 3, 4, 5). A distinct feature of mitochondrial fusion is the sequential fusion of two pairs of bilayers. Key questions regarding the spatiotemporal control of these events remain. Specifically, how are the activities of the outer and inner membrane fusion GTPases, mitofusin 1/2 (Mfn1/2), and optic atrophy 1 (Opa1), respectively, coordinated and regulated?

Solute carrier family 25 member A46 (SLC25A46) interacts with both fusion GTPases and has emerged as an intermembrane bridging protein, playing roles in regulating mitochondrial membrane dynamics (6, 7, 8, 9). SLC25A46 is a member of the solute carrier family 25 of mitochondrial transporters, which transport substrates such as nucleotides, vitamins, amino acids, and fatty acids across the inner mitochondrial membrane (10). Residing in the outer mitochondrial membrane (OMM), SLC25A46 lacks the signature sequence motifs of the SLC25 family (10), does not have an identified substrate and contains a distinct N-terminal extension. Mutations in SLC25A46 result in optic atrophy spectrum disorder, Leigh syndrome, and pontocerebellar hypoplasia (11, 12, 13, 14, 15, 16, 17, 18, 19, 20, 21, 22, 23, 24, 25).

In SLC25A46 KO and knockdown models of human neurological disease, mitochondria are often enlarged and aggregated in nervous tissues, exhibiting detached, vesiculated cristae (22, 25, 26, 27, 28, 29). Respiratory chain complex activity is impaired in these models (26, 27), and ATP levels are reduced (27, 28). In human cell lines, knockdown of SLC25A46 induces a tubular mitochondrial network whereas overexpression results in mitochondrial fragmentation (6, 9, 25), suggesting a role in downregulating fusion. The phenotypes of SLC25A46 KO varied depending on the cell lines tested, displaying mitochondrial fragmentation in human fibroblasts and HeLa cells (8), and hyperfilamentous network in other cell lines (30, 31). KO of the yeast ortholog Ugo1 (∼22% sequence identity) also induced fragmentation (32, 33, 34). These observations argue for a role of SLC25A46 in regulating mitochondrial fusion where SLC25A46 levels at the OMM are critical. In fibroblasts and induced pluripotent stem cells-derived cerebral neurons, SLC25A46 is focally present at tips and branches of mitochondrial tubules and at sites of fusion and fission (8). Interestingly the SLC25A46 puncta were proximal to Opa1 in human fibroblasts (8), suggesting spatially defined interactions with SLC25A46 play a role in fusion regulation.

Understanding the molecular details of the SLC25A46-Mfn2-Opa1 interactions can provide constraints on how SLC25A46 organizes sites for mitochondrial membrane remodeling and inform mechanisms for coordinated OMM/inner mitochondrial membrane fusion. In yeast, Ugo1 physically interacts with Fzo1 and Mgm1, the orthologous fusion GTPases (34, 35, 36, 37). Previous studies indicate Opa1 and Mfn2 interact with SLC25A46 (6, 7, 8, 9); however, the specific interfaces mediating these interactions were undefined. In this study, we used crosslinking-mass spectrometry and AlphaFold 2 (https://colab.research.google.com/github/deepmind/alphafold/blob/main/notebooks/AlphaFold.ipynb) modeling to identify the protein-protein interaction surfaces of SLC25A46-Opa1 and SLC25A46-Mfn2. We validate this newly identified SLC25A46-Opa1 interface by mutagenesis and demonstrate a role for this interface in mitochondrial network maintenance.

## Results

### SLC25A46 interacts with Opa1 *via* its bundle signaling element

**Figure 1:**
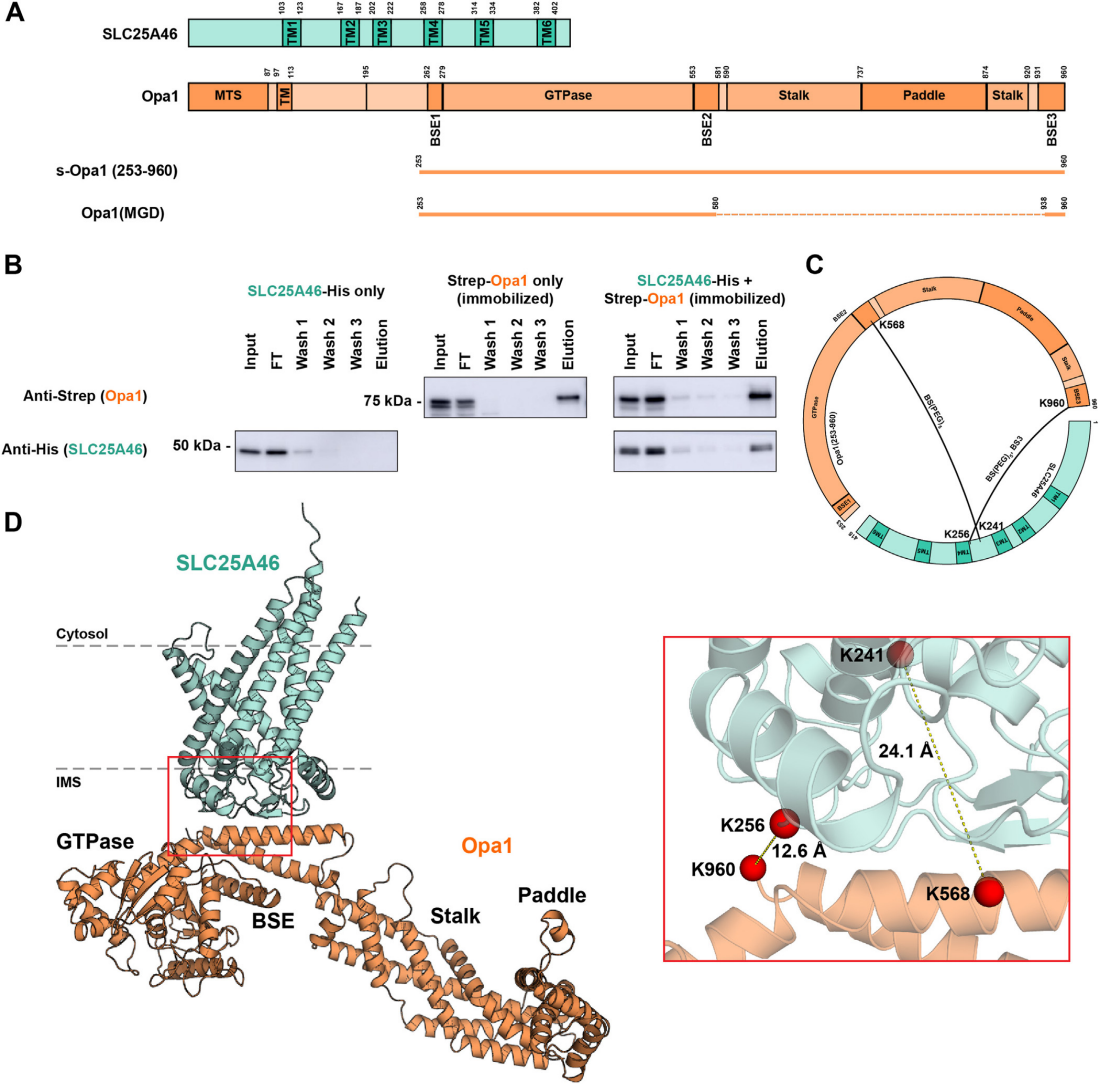
**SLC25A46 interacts with the bundle signaling element (BSE) of Opa1.***A*, domain diagrams of SLC25A46 and Opa1. s-Opa1 (amino acids 253-960) and Opa1(MGD) (minimal GTPase domain comprising the GTPase domain and three BSE helices) are shown as *line* diagrams below full length Opa1 sequence. *B*, GDN extracts prepared from *Pichia pastoris* expressing SLC25A46-His, Strep-Opa1, or coexpressing SLC25A46-His and Strep-Opa1 subjected to StrepTactin column binding and elution. *C*, cross-links identified in BSE helices 2 and 3 of Opa1. Circular map of SLC25A46 and Opa1 with identified lysine-lysine crosslinks (*black arcs*). Crosslinker used to generate the crosslink is indicated. *D*, AlphaFold 2 model of the SLC25A46-Opa1 interaction. N-terminal region of SLC25A46 comprising residues 1 to 83 (disordered) not displayed, residues 1 to 263 of Opa1 not modeled. *Box* region displaying identified cross-links and the calculated Cɑ-Cɑ distances based on the model. GDN, glyco-diosgenin; Opa1, optic atrophy 1; SLC25A46, solute carrier family 25 member A46.

**Supporting Figure 1:**
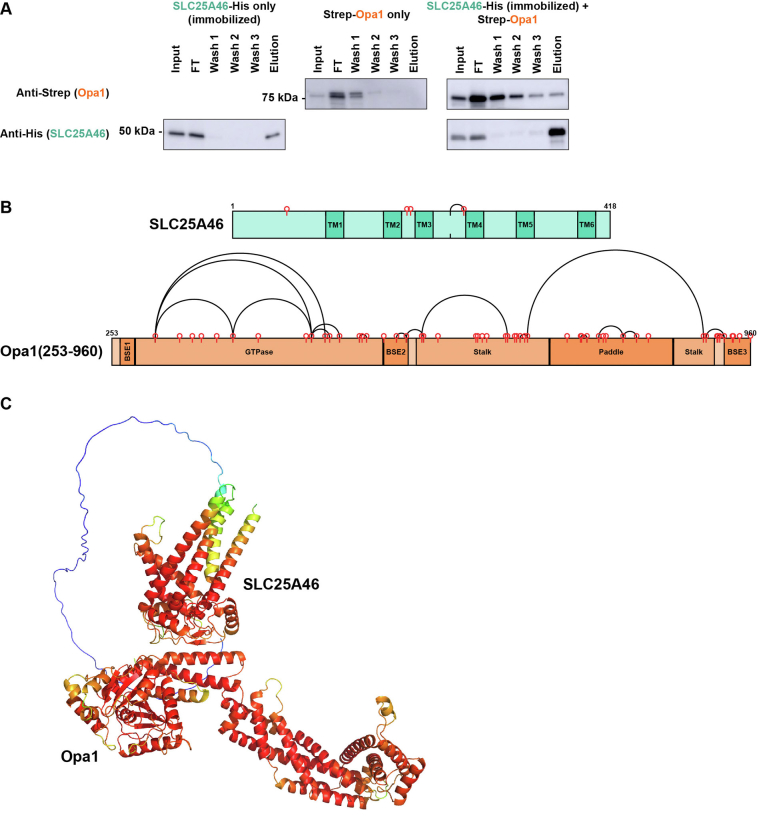


Previous studies used crosslinking, immunoprecipitation, and mass spectrometry to identify an interaction network of Opa1, Mfn1/2, and SLC25A46 (6, 7, 8, 9). We overexpressed human SLC25A46 with Opa1 in *Pichia pastoris* under the control of a strong promoter (alcohol oxidase) (Fig. 1*A*) and found that detergent (glyco-diosgenin, GDN)-solubilized SLC25A46 in *P. pastoris* lysate interacts with column-immobilized Opa1 from lysate in the absence of crosslinkers (Fig. 1*B*). Likewise, column-immobilized SLC25A46 was also found to interact with Opa1 (Fig. S1*A*).

To identify the specific interface responsible for the SLC25A46-Opa1 interaction, we cross-linked the purified complex for analysis by mass spectrometry. Using bis(sulfosuccinimidyl)suberate (BS3) we identified a cross-link between K241 of SLC25A46 and K568 of Opa1 bundle signaling element (BSE) helix 2. Intralinks consistent with folded Opa1 secondary structure elements were identified (Fig. S1*B*). A second cross-linker Bis-N-succinimidyl-(pentaethylene glycol) (BS(PEG)5) with a larger spacer length also cross-linked K241(SLC25A46)-K568(Opa1), and identified a second crosslink between K256 of SLC25A46 and the K960 of Opa1 BSE helix 3 (Fig. 1*C*). We modeled this interaction using Alphafold 2 (38, 39), which consistently revealed a high-confidence and robust interface mediated by the Opa1 BSE, with a topology consistent with the proteins’ suborganellar localization (6, 22, 25, 40, 41) (Figs. 1*D*, and S1*C*).

### Validation of SLC25A46:Opa1-BSE interaction

**Figure 2:**
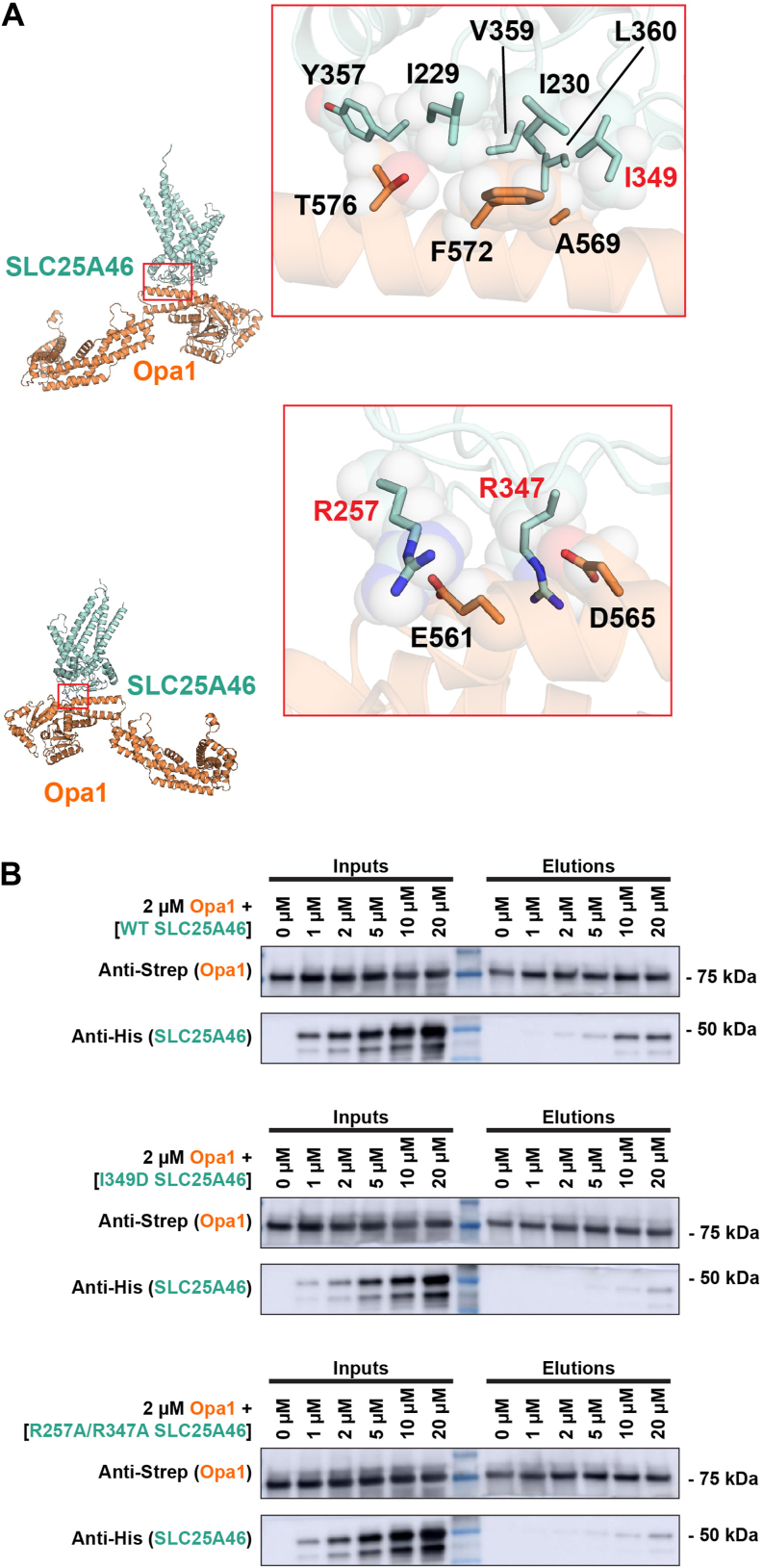
**SLC25A46 mutations in the binding interface diminish binding to Opa1.***A*, SLC25A46-Opa1 interface observed in AlphaFold 2 model. *Top* view: adjacent to the salt bridges is a cluster of hydrophobic residues comprising of I229, I230, I349, Y357, V359, and L360 of SLC25A46 and T576, F572, and A569 of Opa1. *Bottom* view: predicted salt-bridge interactions between R257 of SLC25A46 and E561 of Opa1, and R347 of SLC25A46 and D565 of Opa1. Interface residues highlighted. Mutated residues indicated in *red*. *B*, purified SLC25A46-His WT, I349D mutant or R257A/R347A double mutant were incubated with 2 μM of Strep-Opa1 and subjected to StrepTactin binding and elution experiments. Input and elution analyzed by Western blotting. Opa1, optic atrophy 1; SLC25A46, solute carrier family 25 member A46.

**Supporting Figure 2:**
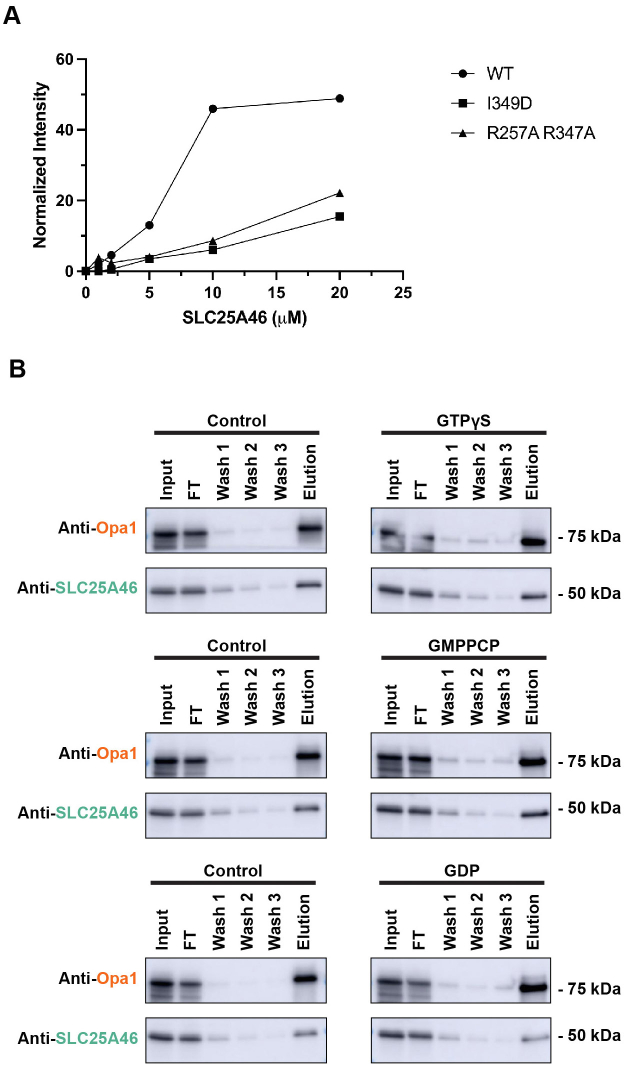


Examination of the SLC25A46-Opa1 interface in the AlphaFold 2 model revealed two salt bridges comprising residues R257 (SLC25A46) and E561 (Opa1); R347 (SLC25A46) and D565 (Opa1). Adjacent to the salt bridges, a cluster of hydrophobic residues, I229, I230, I349, Y357, V359, and L360 in SLC25A46 and A569, F572, and T576 in Opa1 BSE helix 2 appear to further stabilize the interaction (Fig. 2*A*). We tested this newly identified SLC25A46-Opa1 interface by mutating the salt bridges and hydrophobic site residues in SLC25A46 and evaluating if the interaction with Opa1 was maintained. While recombinantly expressed and purified WT SLC25A46 bound column-immobilized Opa1 (consistent with observations in lysates), both the I349D mutation and the R257A/R347A double mutation diminished the interaction (Figs. 2*B* and S2*A*).

The BSE is essential for transducing GTP-hydrolysis stimulated activities of dynamin related protein (42, 43). In dynamin, the BSE frequently moves closer to the GTPase upon GTP hydrolysis (44, 45, 46). To determine if the nucleotide-state of Opa1 affects its interaction with SLC25A46, we incubated the lysates with GTPγS, GMPPCP, and GDP prior to affinity capture on StrepTactin resin. We found that the presence of nucleotide or nucleotide analogues did not affect coelution, indicating nucleotide-coupled BSE rearrangement does not affect Opa1-SLC25A46 interaction (Fig. S2*B*).

**Supporting Figure 3:**
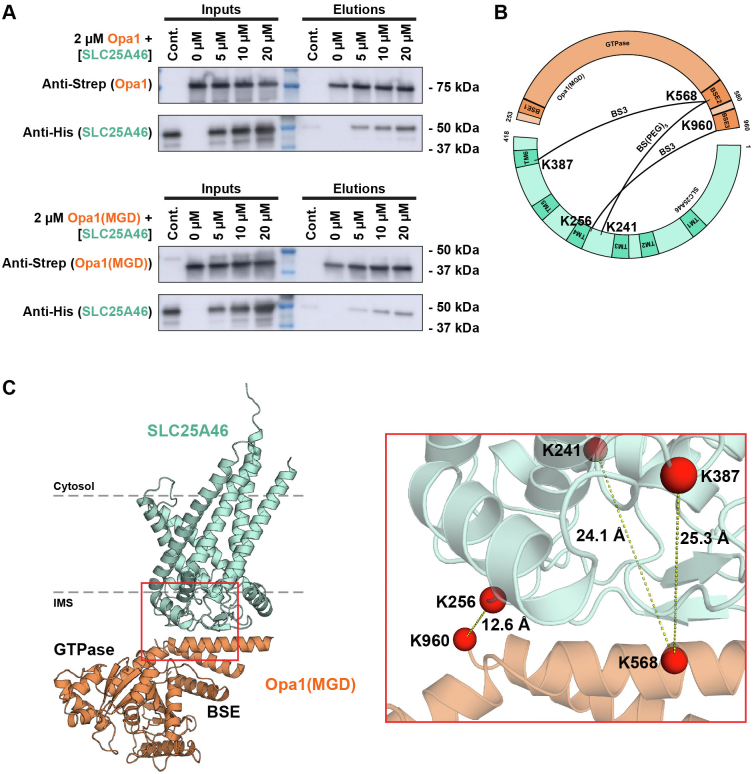


We also generated a minimal construct consisting of the GTPase domain and the BSE alone (Opa1 (minimal GTPase domain, MGD)), to test that the “paddle” and stalk self-assembly regions are dispensable for the Opa1-SLC25A46 interaction (Fig. 1*A*). SLC25A46 coelutes with Opa1(MGD), indicating an Opa1(MGD) construct is sufficient for binding (Fig. S3*A*). We found the same two crosslinks observed for Opa1-SLC25A46 between Opa1(MGD) and SLC25A46. In addition, we observed a third crosslink between K387 of SLC25A46 and K568 of Opa1 in the same vicinity, strengthening our findings (Fig. S3, *B* and *C*).

### Cytosolic facing SLC25A46 interactions with the Mfn2 ectodomain

**Figure 3:**
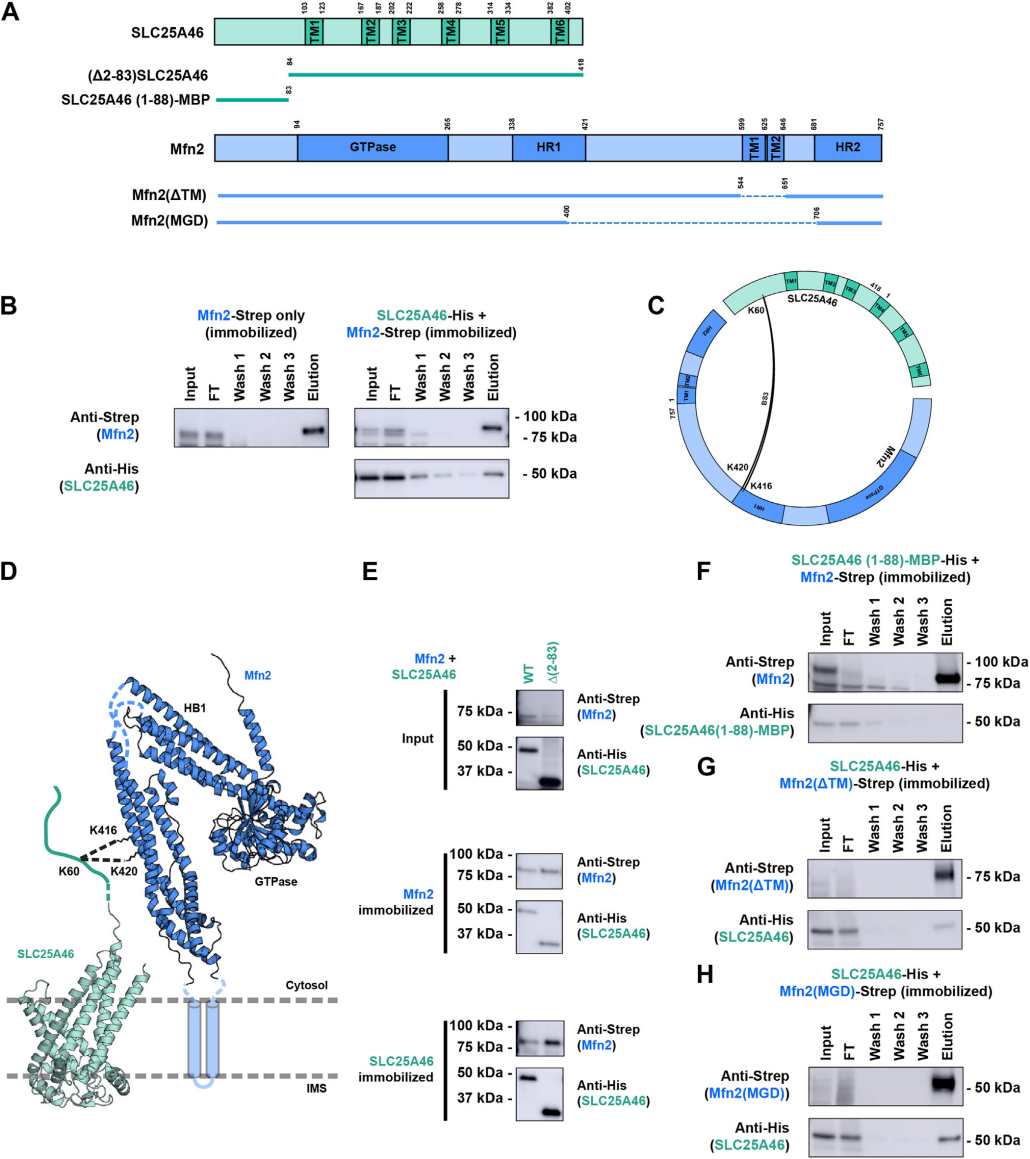
**SLC25A46 interactions with Mfn2.***A*, domain diagrams of full length and truncated constructs of SLC25A46 and Mfn2 are depicted. *B*, SLC25A46-His coelutes upon elution of bound Mfn2-Strep. GDN extracts prepared from *Pichia pastoris* expressing Mfn2-Strep or coexpressing SLC25A46-His and Mfn2-Strep were subjected to StrepTactin column binding and elution. *C*, circular map of SLC25A46 and Mfn2 with the identified lysine-lysine crosslinks (*black arcs*). Crosslinker used is indicated. Cross-links were identified between the cytosol-exposed N-terminal K60 of SLC25A46 and the HR1 lysines K416 and K420 of Mfn2. *D*, model of the SLC25A46-Mfn2 interaction. The identified cross-links are depicted as *dotted lines* in the AlphaFold 2 generated models of SLC25A46 and Mfn2. Helical bundle 1 (HB1) indicated. The N-terminal region of SLC25A46 comprising residues 1 to 83 is predicted to be disordered and is displayed as a hand-drawn cartoon. The transmembrane helices of Mfn2 were poorly modeled and are shown as transparent cylinders, to indicate uncertainty. *E*, (Δ2-83)SLC25A46-His coelutes upon elution of bound Mfn2-Strep. GDN extracts prepared from *P. pastoris* expressing Mfn2-Strep and coexpressing SLC25A46-His or (Δ2-83)SLC25A46-His were subjected to StrepTactin column binding and elution. *F*, residues 1 to 88 of SLC25A46 fused to MBP and His tag at the C terminus (SLC25A46 (1-88)-MBP-His) does not coelute with immobilized Mfn2-Strep. *G*, a Mfn2 lacking the two transmembrane segments (Mfn2 (ΔTM)-Strep) coelutes with SLC25A46-His. *H*, a minimal Mfn2 construct comprising helical bundle 1 (HB1) and the GTPase domain coelutes with SLC25A46-His. GDN, glyco-diosgenin; HR1, heptad repeat 1; SLC25A46, solute carrier family 25 member A46; TM, transmembrane; Mfn, mitofusin 2.

**Supporting Figure 4:**
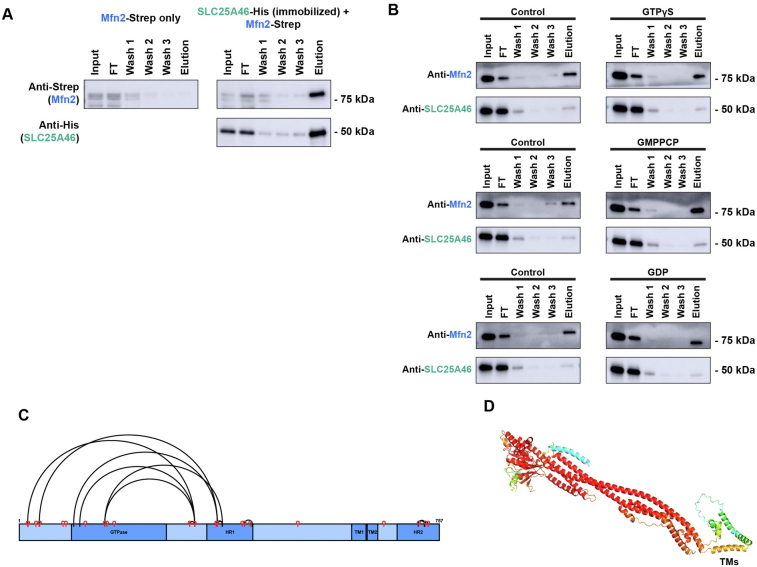


We next investigated if SLC25A46 also interacts with the outer membrane fusion protein Mfn2. We coexpressed the two proteins in *P. pastoris* (Fig. 3*A*) and subjected the GDN-solubilized lysates to column-binding experiments. SLC25A46 coelutes upon elution of column-immobilized full-length Mfn2 (Fig. 3*B*), and Mfn2 coelutes with resin bound SLC25A46 (Fig. S4*A*). To test the SLC25A46-Mfn2 interaction was nucleotide-state dependent, we treated lysates with GTPγS, GMPPCP, or GDP during detergent extraction. The presence of these nucleotides did not enhance or diminish interaction of Mfn2 with SLC25A46 (Fig. S4*B*).

To gain insight into the nature of the SLC25A46-Mfn2 interaction, we performed crosslinking and mass-spectrometry on the purified complex. In addition to intramolecular crosslinks consistent with folded Mfn2 (Fig. S4*C*), two interlinks were identified—between K60 residue of SLC25A46 and residues K416 and K420 of Mfn2 (Fig. 3*C*). K60 of SLC25A46 is part of the first ∼83 residues that are predicted to be disordered in the cytosol (Fig. 3*D*). Residues K416 and K420 are part of the cytosol facing heptad repeat 1 (HR1) of Mfn2 (Fig. 3*D*). In contrast to the Opa1-SLC25A46 models, AlphaFold 2 predicted interface was low confidence for the SLC25A46-Mfn2 complex, particularly for the N-terminal region of SLC25A46, and the Mfn2 transmembrane segments (Fig. S4*D*).

Since cross-links were observed at K60 in SLC25A46, we tested the role of these N-terminal residues in binding Mfn2. We deleted the first 82 amino acids of SLC25A46 termed (Δ2-83) SLC25A46, and coexpressed this protein with Mfn2 in *P. pastoris*. Interestingly, (Δ2-83) SLC25A46 still coeluted with Mfn2 in the affinity capture experiments (Fig. 3*E*), suggesting interactions outside this N-terminal segment mediating Mfn2 interactions. Consistent with this idea, MBP-fusion of the SLC25A46 N terminus (SLC25A46 (1-88)-MBP) was insufficient to capture Mfn2 (Fig. 3*F*). To exclude the possibility that the transmembrane segments of Mfn2 interact with SLC25A46, we coexpressed SLC25A46 with Mfn2 lacking the transmembrane helices (Mfn2 (ΔTM)). We observed coelution of SLC25A46 with Mfn2 (ΔTM) (Fig. 3*G*), supporting an interaction between the Mfn2 ectodomain and cytosol-exposed regions of SLC25A46. Expression of a minimal Mfn2 (MGD) construct, containing only the helical bundle 1 and GTPase domain (47), still bound SLC25A46, suggesting this region of the ectodomain interacts with the SLC25A46 cytosolic face (Fig. 3*H*).

### Mutations disrupting SLC25A46-Opa1 interaction alter mitochondrial morphology

**Figure 4:**
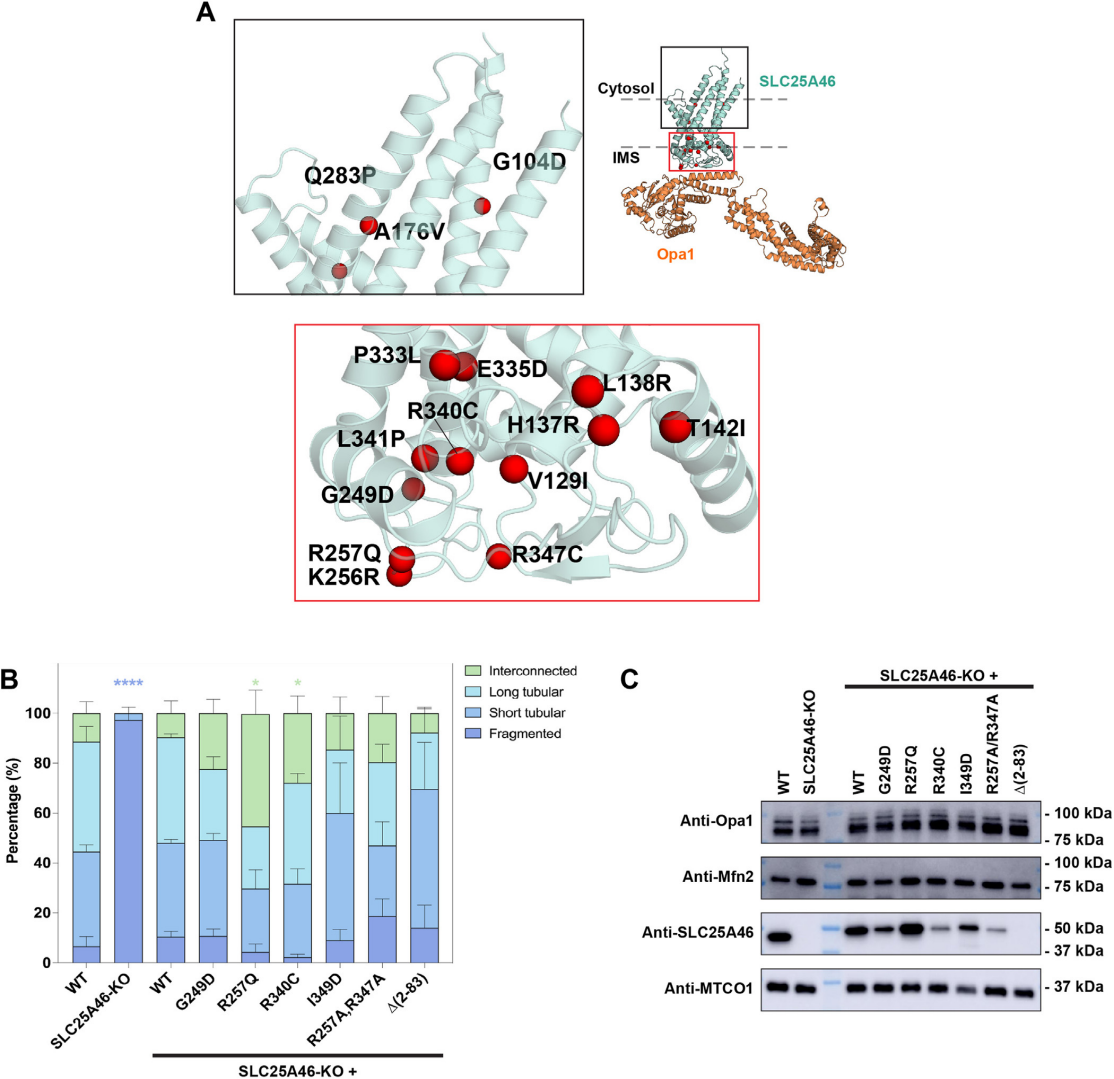
**Mutations disrupting Opa1 interaction alter mitochondrial morphology.***A*, identified SLC25A46 disease mutations mapped on AlphaFold 2 model of SLC25A46-Opa1 complex. *Box* regions enlarged on the *right* to display the mutations. *B*, graph bar representing the relative proportion of scored mitochondrial morphologies in HCT116 WT, SLC25A46-KO, or SLC25A46-KO cells expressing SLC25A46 variants. Mitochondrial morphology for 300 cells were scored for each condition and evaluated (see Materials and Methods). Significance of difference is tested relative to WT using unpaired *t* test with Welch’s correction. *Blue* ∗∗∗∗: *p* < 0.0001 when compared to scored WT cells showing fragmented mitochondria. *Green* ∗: *p* < 0.05 when compared to scored WT cells showing interconnected mitochondrial network. *C*, Western blot of expression levels for Opa1, Mfn2, SLC25A46, or MTCO1 in HCT116 WT, SLC25A46-KO, or SLC25A46-KO cells expressing SLC25A46 variants. (Δ2-83)SLC25A46 not detected as commercially available antibodies detect the N-terminal epitope of SLC25A46. MTCO1 is used as loading control. Mfn, mitofusin 2; Opa1, optic atrophy 1; SLC25A46, solute carrier family 25 member A46.

A cluster of disease mutations reported in SLC25A46 face the intermembrane space (Fig. 4*A*) (16). While many mutations were reported to destabilize the protein, certain disease-causing mutations (T142I and R340C) cause a hyperfused mitochondrial phenotype (6, 8, 9, 16, 25), similar to that observed with siRNA/shRNA knockdown of SLC25A46 (6, 9, 25).

To investigate cellular mitochondrial phenotypes upon SLC25A46 perturbations, we imaged mitochondrial networks in the human colorectal carcinoma cell line HCT116. Under steady-state conditions, WT and SLC25A46 KO HCT116 cells demonstrate similar mitochondrial network morphology. Previous studies show that nutrient starvation triggered stress can induce mitochondrial hyperfusion (48, 49). To test if mitochondria in HCT116 cells undergo similar stress-induced change, cells were grown in serum-free medium. Under such conditions, we observed significant mitochondrial fragmentation in SLC25A46 KO cells but not in WT cells. Reintroduction of WT SLC25A46 rescued this phenotype (Fig. 4*B*, Supporting Video 1), consistent with previous observations in human fibroblasts and HeLa cells (8). We found that expression of R257Q or R340C SLC25A46 mutants induced interconnected mitochondrial networks, also in agreement with previous studies (8, 25) (Fig. 4*B*, Supporting Video 1). The mitochondrial morphology in G249D-expressing cells was similar to WT (Fig. 4*B*, Supporting Video 1). Expression levels of R340C and G249D mutants never reached WT-like levels (Fig. 4*C*), likely due to protein instability (6, 8, 9, 16, 22).

We next tested the effects of the newly identified Opa1-SLC25A46 interactions on mitochondrial morphology. Expression of I349D SLC25A46 (disrupts a hydrophobic interface between the Opa1 BSE) produced shorter mitochondrial tubules compared to WT, whereas R257A/R347A mutant (disrupt SLC25A46-Opa1 salt bridge interactions) resulted in more fragmented mitochondria (Fig. 4*B*, Supporting Video 1). These results suggest that the identified SLC24A45-Opa1 interaction interface is important for maintaining a tubular mitochondrial morphology. Expression of the (Δ2-83)SLC25A46 resulted in an increase in short mitochondrial tubules (Fig. 4*B*, Supporting Video 1). In these experiments, we did not observe changes in levels of Mfn2 or Opa1 due to SLC25A46 knockout or expression of varying levels of mutant SLC25A46 (Fig. 4*C*). The expression level of (Δ2-83)SLC25A46 could not be evaluated due to the inability of commercial antibodies to detect this mutant (Fig. 4*C*).

**Supporting Figure 5:**
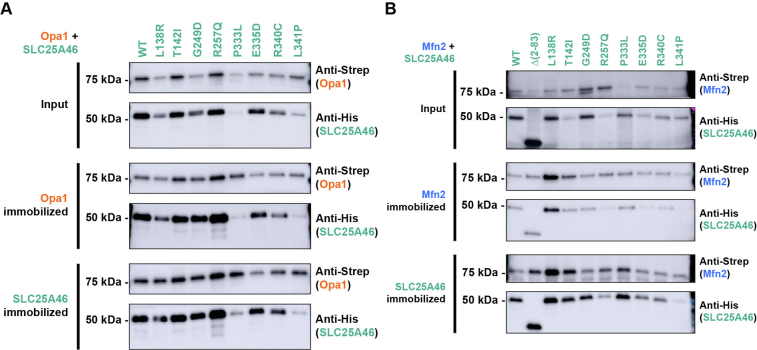


We also investigated SLC25A46 disease mutants expressed in yeast. Under these conditions, many of SLC25A46 mutants still bound Opa1 (Fig. S5*B*). We also saw little effect of patient-derived mutations on the Mfn2-SLC25A46 interaction (Fig. S5*B*). We note that in these binding experiments we were unable to achieve similar expression levels of the respective proteins, and binding may only describe one aspect a complex phenotype only observable under cellular conditions.

## Discussion

Mitochondria undergo morphological changes tightly coupled to cellular physiology (4). While the identity of the GTPases responsible for sequential mitochondrial fusion have long been identified (50), and mechanisms for individual fusion steps have been described (51, 52, 53, 54), questions remain regarding how outer and inner-membrane activities are coordinated and regulated. Emerging evidence points to SLC25A46 as a regulator of fusion activity, with protein levels influencing the fusion-fission balance (6, 8, 25). Consistent with this role, SLC25A46 is observed to localize to the fusion sites (8). In this study, we investigate the physical interaction between SLC25A46 and the membrane fusogens Opa1 and Mfn1/2. Combining crosslinking mass-spectrometry, and AlphaFold 2 modeling, we identify and validate the BSE region in Opa1 interaction with the intermembrane space-face of SLC25A46. We also present evidence for an interaction between SLC25A46 cytosolic face and the Mfn2 ectodomain. The Mfn2 region we identify includes helical bundle 1 and GTPase domains, but not the transmembrane segments. This is consistent with yeast studies, where residues 1 to 294 of Ugo1 interact with Fzo1 in a region comprising residues N-terminal to the first transmembrane helix and the C-terminal helical repeat 2 (34).

Reports from several studies indicate that mitochondrial morphology is sensitive to SLC25A46 levels. Schuettpelz *et al.* reported mitochondrial fragmentation in human fibroblasts and HeLa cells upon SLC25A46 KO (8, 55). In SLC25A46 KO HCT116 cells, we observed mitochondrial fragmentation upon serum starvation. In addition, we observed a ∼2-fold slower growth of this cell line, consistent with observations in SLC25A46 deleted fibroblasts (8). In previous studies, overexpression of SLC25A46 increased mitochondrial fragmentation, suggesting that at high levels, SLC25A46 may downregulate the activities of the protein fusogens (6, 25, 31). One possible mode of inhibitory action may be SLC25A46 binding resulting in steric competition for Opa1 self-assembly, which relies on the BSE and stalk regions to mediate formation of helical filaments (56, 57, 58). Consistent with this prediction, levels of Opa1 oligomeric complexes were found to increase upon loss of SLC25A46 (8).

In our study, SLC25A46 mutants targeting Opa1 interaction did not promote a hyperfused phenotype, but instead disrupted the mitochondrial network. This suggests that a SLC25A46-Opa1 interaction promotes fusion necessary for a tubular network. Although we could not estimate the relative levels of (Δ2-83)SLC25A46, this N-terminal deletion reduced the filamentous network, while increasing the proportion of short tubules, also suggesting a fusion defect. Since the SLC25A46 N-terminal region alone is insufficient to bind with Mfn2, we speculate that this region may play a stabilizing role facilitating interactions of additional cytosolic-facing regions with the ectodomain of Mfn2. Taken together, these observations provide evidence for SLC25A46 interactions playing an activating role in fusion when expressed at physiological levels.

Our results provide a basis for future functional analyses of SLC25A46 regulation of the fusion machinery. SLC25A46 protein-protein interactions also may regulate cristae morphology, which is dramatically affected upon loss of SLC25A46 (6, 8, 25, 26, 27, 29). Proteomics studies have also implicated SLC25A46 endoplasmic reticulum interaction partners in maintaining endoplasmic reticulum-mitochondria contacts facilitating lipid flux (6, 7, 8). Future mechanistic structure-activity studies are necessary to explore these other roles of SLC25A46 in cristae regulation and lipid metabolism.

## Experimental procedures

### Plasmids

Genes for SLC25A46, Mfn2, Opa1(253-960), and Opa1(MGD) constructs were synthesized (GenScript) and cloned into pPICZ A for *P. pastoris* expression. C terminus of SLC25A46 was fused to a (G_4_S)_3_ linker followed by tobacco etch virus cleavage site and deca-histidine. N-terminus of Opa1(253-960), Opa1(263-960), Opa1(MGD) was tagged with Twin-Strep tag followed by HRV 3C cleavage site and (G_4_S)_3_ linker. The C-terminus of Mfn2 was fused to G_3_S linker, HRV 3C cleavage site and Twin-Strep tag. For coexpression, SLC25A46 and either Mfn2 or Opa1 with tags with its own AOX1 promoter and terminator were cloned into the same plasmid. (SLC25A46 (1-88)-MBP) was generated from SLC25A46 (1-88) fused to MBP followed by a GGGS linker tobacco etch virus site and His10 tag. Mfn2 (ΔTM) was generated from Mfn2 (2-544)-GGS linker-Mfn2 (651-757). Mfn2 (MGD) was generated from Mfn2 (2-400:706-757). Strep-Opa1 (253-960) and Strep-Opa1 (MGD) were also cloned into pET-28a (+) vector for expression in *Escherichia coli*.

### Generation of *Pichia* strains

Expression strains were generated from *P. pastoris* strain SMD1163 (*his4 pep4 prb1*) (kind gift from Dr Tom Rapaport (Harvard Medical School) according to established protocols (59). Plasmids were transformed into SMD1163 by electroporation and grown on YPDS (1% yeast extract, 2% peptone, 2% dextrose, 1 M sorbitol) zeocin (InvivoGen, ant-zn-1p) plates. 7 to 14 resulting colonies were grown in BMGY (1% yeast extract, 2% peptone, 100 mM potassium phosphate pH 6.0, 1.34% yeast nitrogen base, 4 × 10^−5^% biotin, 1% glycerol) and induced in BMMY (replaced glycerol with 0.5% methanol in BMGY). Cultures were screened for protein expression by Western blot using anti-His, anti-Strep, anti-SLC25A46, anti-Opa1, or anti-Mfn2 antibodies.

### Binding experiments using lysates

*P. pastoris* (1 L) expressing SLC25A46, Opa1, Mfn2 alone or in combination were harvested and cryo-milled using SPEX SamplePrep 6875D Freezer/Mill. For StrepTactin column-binding and elution, milled cells (125 mg) were resuspended in 3 ml of 25 mM Hepes, 150 mM NaCl, pH 8.0 buffer containing 1% GDN and protease inhibitors, tumbled for 1-1:30 h at 4 °C. Milled cell input was increased to 375 mg (3× original input) when needed for increased signal. Suspension was centrifuged at 20,000 for 30 min at 4 °C. Supernatant (100 μl) was incubated with 50 μl of StrepTactin XT 4Flow high-capacity resin in a spin column (Abcam, ab286861) at 4 °C for 15 min followed by centrifugation at 500*g* for 30 s. Resin was washed 3× with 100 μl buffer containing 0.1% GDN. To elute, the resin was incubated with 100 μl buffer with 50 mM biotin for 15 min and centrifuged. Same procedure for Ni-NTA binding experiments except that the binding and elution buffer contained 80 mM and 500 mM imidazole, respectively.

### AlphaFold 2 modeling

Protein sequences for human SLC25A46, Opa1, and Mfn2 were run through in a AlphaFold2 Google ColabFold – v 1.5.3 workbook (https://colab.research.google.com/github/deepmind/alphafold/blob/main/notebooks/AlphaFold.ipynb) (38, 39) (https://colab.research.google.com/github/deepmind/alphafold/blob/main/notebooks/AlphaFold.ipynb). Five independent runs generated the top three ranked relaxed models for SLC25A46-Opa1 and SLC25A46-Mfn2 models, respectively.

## Data availability

Mass spectrometry data available on request.

## Supporting information

This article contains supporting information (60, 61).

## Conflict of interest

L. H. C. is an advisor for Stealth Biotherapeutics. The other authors declare that they have no conflicts of interest with the contents of this article..

## Supporting information

Supporting Figures Caption
